# Ion Conduction and Its Activation in Hydrated Solid Polyelectrolyte Complexes

**DOI:** 10.3390/polym9110550

**Published:** 2017-10-25

**Authors:** Souvik De, Annika Ostendorf, Monika Schönhoff, Cornelia Cramer

**Affiliations:** 1 NRW Graduate School of Chemistry, University of Muenster, Wilhelm-Klemm-Str. 10, D-48149 Muenster, Germany; souvikiitm@gmail.com; 2 Institute of Physical Chemistry, University of Muenster, Corrensstraße 28/30, D-48149 Münster, Germany; Annika-Ostendorf@gmx.de (A.O.); schonhoff@uni-muenster.de (M.S.)

**Keywords:** polyelectrolyte complexes, temperature-dependent ionic conductivity, relative humidity, water content, activation enthalpy, time-temperature superposition principle, conduction mechanism

## Abstract

For the first time, temperature-dependent conductivities at constant water content for a series of solid polyelectrolyte complexes with varying mixing ratios of anionic poly(sodium 4-styrene sulfonate) and poly(diallyldimethylammonium chloride) are presented. For water absorption, the samples are first equilibrated at an ambient temperature and at fixed relative humidity (RH). During the conductivity measurements, the so achieved water content of the samples is kept constant. At all of the hydration levels, the dc conductivities of the hydrated polyelectrolyte complexes (PEC) display Arrhenius behavior with activation enthalpies that are significantly lower than those of dry complexes. The activation enthalpy decreases linearly with water content. The lower activation enthalpies in case of hydrated as compared to dried complexes are attributed to a lowering of the energy barriers for ion motion. Finally, it is shown that the temperature-dependent conductivity spectra at constant water content obey the time-temperature superposition principle. Additionally, temperature-dependent conductivities at constant water content are compared to data sets determined in a separate study with constant RH at all of the temperatures. For the latter case, the influence of the type of alkali ion is also considered. Using the broad variety of data sets, the influences of water content and temperature on the conductivity mechanism can be separated from each other.

## 1. Introduction

Initiated by the work of Bungenberg de Jong and Kruyt [1], polymer networks formed by complexation of oppositely charged polyions have been subject of early research. Apart from mutual Coulomb interactions between the polycations and polyanions [2], the major driving force for the formation of polyelectrolyte complexes (PEC) is the increase in entropy due to liberation of small counterions [3]. However, interactions such as hydrogen bonding and hydrophobic interactions may also play an additional role in the formation of PEC [4]. More recently, the precipitation reaction occurring between oppositely charged polyelectrolytes in solution due to strong electrostatic interactions has moved into focus because of their potential application in medicine [5], or in energy storage devices such as fuel cells [6,7,8]. Additionally, PEC have gained considerable attention due to their similarities to polyelectrolyte multilayers (PEM), made from layer-by-layer self-assembly, in terms of transport properties and complexation on a microscopic scale [9,10]. Moreover, the phase diagrams of PEM and PEC show enough resemblance to consider the latter as a model system for PEM [11].

During the past years progress has also been made in the theoretical understanding of PEM and PEC by developing and applying different types of computer simulation techniques [12,13]. Holm and coworkers showed for mixtures of poly(sodium 4-styrene sulfonate) (PSS) and poly(diallyldimethylammonium chloride) (PDADMAC) with water and salt ions included, that water molecules in such mixtures are strongly bound and move significantly slower than in free water. This was deduced from a significant decrease of the dielectric constant and a reduced diffusion coefficient [12].

Systematic studies on the ion transport in PEM and PEC systems are still rare. Durstock and Rubner [14] were the first who reported on dc conductivities of PEM films in the range from 10^−12^ S cm^−1^ to 10^−7^ S cm^−1^. Work on other PEM materials followed [15,16,17]. Although e.g., Farhat et al. [18] and more recently also Ghostine et al. [19] showed the presence of salt ions in PEM, a systematic conductivity study over a broad range of relative humidity (RH) in PEM by Akgöl et al. confirmed that protons are the major conducting species in multilayers [20]. Nevertheless, PEM with tunable conductivity are difficult to obtain. The reason for this is that PEM are formed by self-organization and the amount of incorporated ions is, therefore, hard to control.

On the other hand, systematic investigations of the influence of polyion stoichiometry on the ion transport properties are feasible for PEC, since a predefined amount of small ions can be incorporated via controlling of the mixing ratio of polyanions to polycations. In spite of the advantage of known stoichiometry, only a few studies have been made to explore the ion dynamics of PEC, see for example the review in Reference [21]. In the 1960s, Michaels et al. [22,23] reported on the dielectric properties of solid hydrated PEC, exposed to elevated relative humidities. In the following years, research on polyelectrolyte complexes mainly focused on soluble complexes in aqueous medium, see e.g., Reference [24]. Newer work on PEC is reviewed in Reference [25].

The lack of extensive studies on the ion dynamics in precipitated PEC is probably due to the low dc conductivity of dry PEC. Imre et al. have reported conductivity data of dry PEC and concluded that Na^+^ ions are dominating the ion transport [26,27]. Later, a systematic conductivity study by De et al. on hydrated PEC with Na^+^ or Cs^+^ as counterions showed that therein hydrated alkali ions are the major conducting species [28]. Moreover, with the help of a recently developed scaling concept, which is based on conductivity spectra recorded at constant RH (cRH) [29], the authors have also discussed the role of water molecules in transport properties of hydrated PEC at ambient temperature. It was argued that the relative humidity as a tunable outer parameter influences the activation barriers for the ion transport. However, the effect of temperature in activating ion transport has not yet been explored in humid PEC. Therefore, a deeper insight into the ion dynamics and its activation is still lacking but necessary to understand the microscopic transport phenomena in humid PEC. Temperature-dependent conductivity studies are a powerful tool to explore the microscopic transport properties of ion-conducting materials [30]. *T*-dependent conductivities reveal the activation enthalpy of the macroscopic charge transport, which in turn allows for the interpreting of the underlying microscopic ion dynamics.

For the study presented here, we have prepared several compositions of polyelectrolyte complexes from poly(diallyldimethylammonium chloride) and poly(styrene sulfonate) abbreviated as *x* MPSS·(1 − *x*) PDADMAC throughout the text. “M” stands for Na or Cs, respectively. The samples were allowed to absorb water during equilibration in an environment of defined relative humidity (RH). We have used conductivity spectroscopy to determine the conductivity of different compositions of these humid polyelectrolytes complexes as a function of temperature.

One possibility of studying the temperature dependence of the ion dynamics of hydrated PEC is to vary the temperature while keeping the RH of the environment constant. These results will be referred to as results from the “cRH-series”. However, when interpreting data of the cRH-series, one has to consider the following aspect: If a sample is equilibrated at a particular temperature in an atmosphere of constant RH and then subjected to heating or cooling, the water content in the sample does not necessarily remain fixed, although the relative humidity of the environment is kept constant. The reason for this is that the absolute amount of water in the atmosphere varies with temperature, even though the relative humidity remains constant. Therefore, the water content in PEC at a particular RH might also vary with temperature. Hence, the results of the cRH-series will be mainly presented in the part “Supplementary Materials” and only briefly discussed in one subsection of the main paper.

To separate the effect of temperature on the ion motion from the combined effect of temperature and water content within the samples, the focus in the following sections will be on *T*-dependent spectra recorded at a fixed water content (denoted as cWC-series). For the cWC-series, the samples were first equilibrated at ambient temperature in an environment of fixed relative humidity in which the PEC samples can absorb water. During the temperature-dependent conductivity measurements, this water constant was maintained by isolating the sample within a closed sample cell from the outer atmosphere.

In this contribution we present for the first time temperature-dependent conductivity spectra of hydrated *x* NaPSS∙(1 − *x*) PDADMAC PEC at constant water content. In addition to the comparison of the absolute conductivity values, the variation of the activation enthalpy as a function of PEC composition and water content is also discussed. Furthermore, an effort has been made to understand the combined effects of water content and temperature on the ion dynamics by scaling of the temperature-dependent conductivity spectra to a master curve.

## 2. Materials and Methods

### 2.1. Materials

The anionic polyelectrolyte poly(sodium 4-styrene sulphonate) (NaPSS, average molar mass 70,000 g/mol) was purchased from Acros organics (Geel, Belgium) as a solid white powder. This powder was dissolved in ultrapure water and dialyzed against ultrapure water (filter cut-off: 25 to 30 nm pore size) in order to filter out the short chains and other possible contaminations. After dialysis, the solution was freeze-dried to obtain pure NaPSS.

CsPSS was prepared from NaPSS by an ion exchange technique as explained in Reference [28]. The cationic polyelectrolyte poly(diallyldimethylammonium chloride) (PDADMAC) with a molar mass ranging from (100,000 to 200,000) g/mol was purchased from Sigma-Aldrich GmbH (Steinheim, Germany) as a 20 wt % aqueous solution and used as received.

### 2.2. Sample Preparation

**Figure 1:**
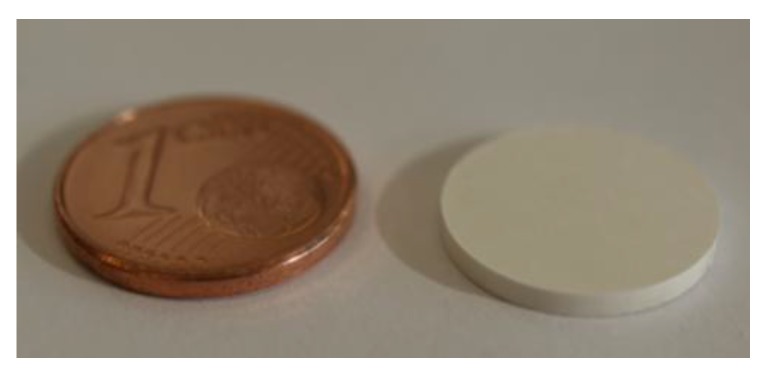
Pressed pellets of the solid polyelectrolyte complexes (PEC) materials before sputtering.

For complex formation, appropriate amounts of 0.05 M aqueous solutions of PDADMAC were added dropwise into a 0.05 M aqueous solution of NaPSS or CsPSS, respectively, and stirred constantly during the complexation reaction. After the complexation, the precipitated complex and the remaining water/salt solution were dialyzed against ultrapure water in order to get rid of the free salt that formed during the complexation reactions. The dialysis was continued until the conductivity of the exchanged water fell below 2 μS cm^−1^. After completion of the dialysis, the complexes were freeze-dried. For conductivity measure ments, cylindrical pellets were prepared from approximately 0.05 g of powdered sample by applying a pressure of 12.5 kN cm^−2^ for 2 min. The so obtained pellets are shown in Figure 1.

The two opposite faces of the cylindrical pellets were sputtered with gold. The pressed samples were equilibrated in an atmosphere of fixed relative humidity. Our previous results from in situ conductivity measurements under humid conditions show that an equilibration time of one week was sufficient to obtain reproducible results [28]. Hence, in the present investigation, samples were equilibrated for one week. A gravimetric analysis as described in Reference [28] was performed to determine the water content.

The stoichiometry of the solid state PEC is given by the ratio of polycations to polyanions. A PEC composition specified as 0.6 NaPSS·0.4 PDADMAC implies that per 0.6 mol monomers of the polyion PSS there are 0.4 mol monomers of the polycation PDADMA. When considering all of the charged groups on the different polyions together, the above composition yields an intrinsic charge compensation of polyion groups of 80%. By contrast, 20% of the charged groups on the polymers (here PSS) are extrinsically charge compensated by Na^+^ ions.

### 2.3. Conductivity Measurements

The investigation of the ion dynamics is performed by frequency-dependent conductivity spectroscopy. This technique has the advantage that the dc conductivity can be extracted from the measured spectra, even in the presence of blocking electrodes. The applied broadband method not only allows for the studying of the macroscopic ion transport, but also of elementary steps of the ion movements [30]. In the measurements, the complex conductivity that consists of a real and an imaginary part, denoted as σ′ and σ″, can be determined. The imaginary part of the conductivity is proportional to the real part of the permittivity. Similar to many complex physical quantities, the real and the imaginary part of the complex conductivity are interconnected via Kramers-Kronig-relations. This implies that they contain the same information and can be transformed into each other, provided that the complete experimental spectrum is known. In this work, both the real and the imaginary part of the complex conductivity were experimentally determined, but the discussion will focus on the real part of the conductivity.

In order to investigate the influence of varying outer conditions on the conductivity of PEC, temperature-dependent admittance studies at constant relative humidity (cRH) were carried out inside a constant climate chamber (HPP 108; MEMMERT GmbH & Co., KG, Schwabach, Germany). The measurements were performed at a relative humidity of 46%, 55% and 64%, respectively, which was always fixed during the temperature variation between 298 K and 333 K. Measurements beyond these ranges of temperature and relative humidity were not possible due to condensation of water vapor inside the chamber. The measurements were performed with an impedance analyzer (hp 4172, Hewlett-Packard, Böblingen, Germany), over a frequency range of 5 Hz to 13 MHz.

For the investigation of PEC samples with constant water content (cWC-series), a closed cylindrical sample cell was used. Temperature-dependent conductivity spectra (between 0.1 Hz and 3 MHz) at fixed water content were determined using an ALPHA-AN-high resolution analyzer equipped with a Quatro cryosystem from Novocontrol Technologies (Montabaur, Germany). Measurements were performed in a so-called sandwich conformation where the sample was placed between a lower and an upper electrode. In case of temperature-dependent conductivity measurements in the cWC-series, the sample was first placed onto the lower electrode within an open sample holder and was kept for humidification inside of a glove box at room temperature. The relative humidity inside the glove box was maintained by appropriate saturated salt solutions as reported elsewhere [28,31]. After the equilibration of the sample at a particular RH, the sample holder was closed inside the glove box, thereby pressing the upper electrode onto the upper face of the sample. The closed sample holder was then removed from the glove box and placed into the Novocontrol measurement cell. Our temperature range extends from 233 K to 313 K with steps of 5 K. We first heated the sample from 293 K to 313 K. This first heating was followed by a couple of cooling and heating cycles between 233 K and 313 K, all yielding reproducible results.

The specific conductivity is obtained by multiplying the experimentally determined admittance with the cell constant, *d*/*A. A* denotes the surface area of the gold electrodes and *d* is the sample thickness measured after sputtering. The bulk resistance was obtained by fitting an equivalent circuit, consisting of a constant phase element connected in parallel with a resistor, to the experimental impedance data. The dc conductivity was obtained by multiplying the inverse of the bulk resistance with the cell constant. More details about the data analysis were already presented in References. [26,28].

## 3. Results

### 3.1. Water Content and Its Influence on the Conductivity

**Figure 2:**
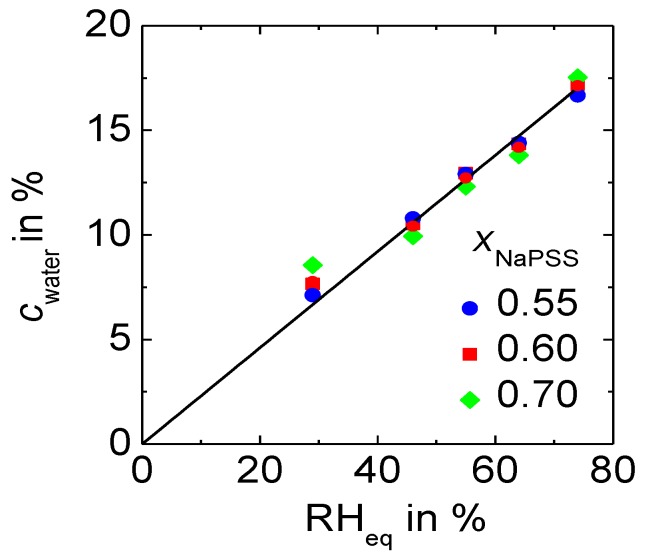
Influence of the relative humidity of the environment on PEC: Water content of *x* NaPSS∙(1 − *x*) PDADMAC at ambient temperature.

As the water content plays a crucial role for the conductivity of polyelectrolyte complexes, we first determined the water content in polyelectrolyte complexes equilibrated at varying relative humidity and at ambient temperature. The range of composition reported here refers only to those complexes with an excess of alkali ions, which are subject of this study. Figure 2 presents the water content, *c*_water_, as a weight fraction determined by gravimetric analysis for *x* NaPSS∙(1 − *x*) PDADMAC PEC samples equilibrated at different RH values (RH < 90%). The values reported here were determined from three consecutive measurements (each separated by a 24 h gap), which showed basically the same mass for the samples with deviations only in 4th or 5th decimal.

In the presented RH range the water content in the complexes does not depend on PEC composition, but remains constant within experimental error. Figure 2 displays a direct proportionality between *c*_water_ and the relative humidity of the environment. A deviation from linearity only occurs at RH > 90% as shown in Reference [28]. Hence, we argue that within the RH-range investigated in this work, the relative humidity at which the samples were equilibrated, ${\mathrm{RH}}_{\mathrm{eq}}$, is a direct measure for the water content in the PEC:

(1) $$c_{\mathrm{water}}=a\cdot {\mathrm{RH}}_{\mathrm{eq}}.$$

The index “eq” has been added to distinguish the relative humidity at which the PEC samples were equilibrated at ambient temperature in the cWC-series from the RH-values of the cRH-series, where the relative humidity of the surrounding air was kept constant at each temperature.

The value of the proportionality factor *a* is determined by the arithmetic mean of the water content at different compositions and it is found to be: *a* = 0.230 ± 0.005. It refers to the relative humidity and the weight fraction of water within the complex both being given in %.

In the following sections, this value is now used to convert all of the measured ${\mathrm{RH}}_{\mathrm{eq}}\;$ values into the water content of the PEC samples of the cWC series. The influence of the type of alkali cation M (Na^+^ or Cs^+^) in *x* MPSS∙(1 − *x*) PDADMAC PEC on the water content in PEC is displayed in Figure S1 of the Supplementary Materials.

**Figure 3:**
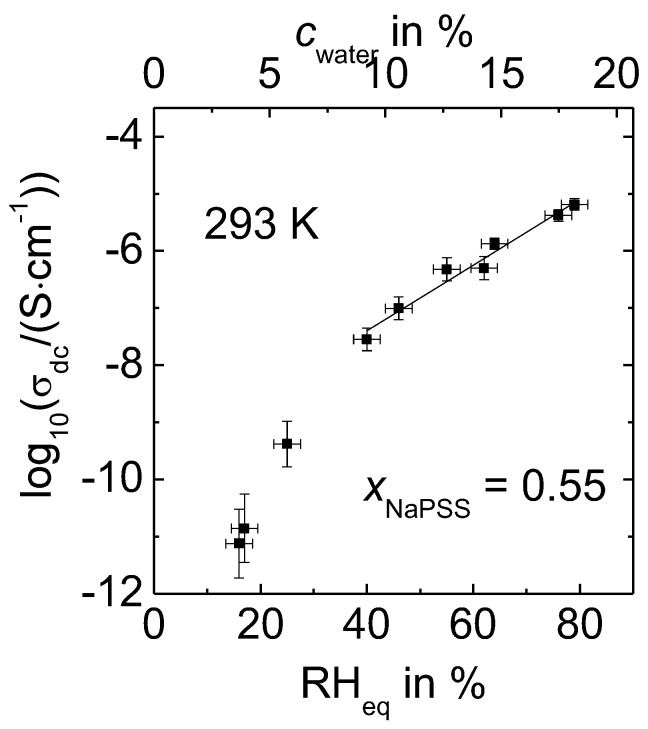
dc conductivities of 0.55 NaPSS∙0.45 PDADMAC determined at 293 K as a function of relative humidity (RH)_eq_ and water content, respectively.

Figure 3 shows dc conductivity data of 0.55 NaPSS∙0.45 PDADMAC at fixed water content obtained from measurements using a closed sample cell. Note that, in contrast to analogous data published in Reference [28], where the conductivities were recorded at slightly varying ambient temperature, the data of Figure 3 were always measured at a fixed temperature of 293 K. Similar to previous reports within Reference [28], conductivity data of PEC that were equilibrated at RH_eq_ ≥ 40% show a linear dependence on RH_eq_. However, our current results at even lower RH reveal that the conductivity is lower than expected from the extrapolation of conductivity data obtained at RH_eq_ ≥ 40%. This deviation from linearity at lower relative humidity was not evident and subsequently not reported earlier, possibly due to the limited RH range investigated [28].

**Figure 4:**
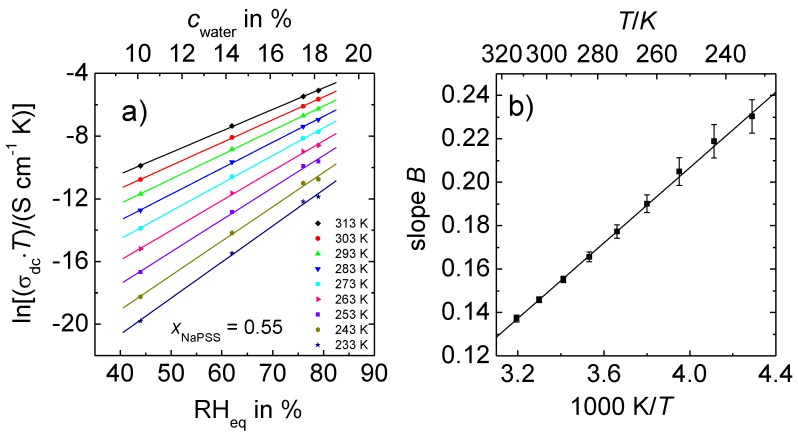
Data of PEC with *x*_NaPSS_ = 0.55 at constant water content. (**a**) ln(σ_dc_ *T*) versus RH_eq_; (**b**) Parameter *B* (slope determined from Figure 4a) as a function of the inverse temperature.

Figure 4a represents the variation of the dc conductivity of 0.55 NaPSS∙0.45 PDADMAC with the relative humidity, at which the samples were equilibrated and the corresponding water content in the PEC, respectively. Note that the data were recorded at RH_eq_ > 40%. Here, the conductivity is high enough so that data can be obtained even well below ambient temperature. The semi-log plot Figure 4a shows a linear relation between $\ln\left(\sigma_{\mathrm{dc}}\cdot T\right)$ and RH_eq_ at all of the temperatures.

The slope of these straight lines is termed *B* and found to decrease with temperature. In fact, as shown in Figure 4b the slope *B* varies linearly with the inverse temperature. This is an important new finding which leads to deeper insights into the influence of water molecules in PEC even well beyond ambient temperature, and it will be discussed in detail later.

**Figure 5:**
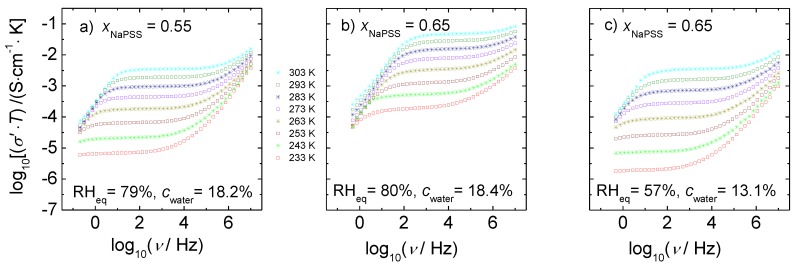
Temperature-dependent conductivity spectra of *x* NaPSS∙(1 − *x*) PDADMAC obtained by conductivity spectroscopy. (**a**) *x*_NaPSS_ = 0.55, RH_eq_ = 79%; (**b**) *x*_NaPSS_ = 0.65, RH_eq_ = 80%, and (**c**) *x*_NaPSS_ = 0.65, RH_eq_ = 57%. The different isotherms were taken at fixed water content as specified in the figures.

Figure 5 shows typical conductivity spectra of PEC for three representative samples of the cWC-series at varying temperatures. Analogous spectra for the cRH-series are shown in Figure S2 (Supplementary Materials). The prime in σ′ characterizes the real part of the complex conductivity. In all of the investigated composition, relative humidity, water content, and temperature ranges, we observe a well-defined plateau where the conductivity is almost independent of experimental frequency. This plateau value can be identified with the dc conductivity.

Apart from the spectra taken at low temperature and low humidity, we also observe a decrease in conductivity at frequencies lower than those of the dc regime. This low-frequency part of the spectra can be attributed to electrode polarization effects that are caused by the presence of blocking electrodes. The frequency at which these polarization effects become visible in the spectra decreases with decreasing temperature and moves out of the experimental frequency window at low temperatures.

At frequencies higher than those of the dc regime, the conductivity increases monotonously with frequency. Moreover, for a particular composition, not only the dc plateau moves to higher conductivity values with increasing temperature, but also the onset of dispersion shifts towards higher frequency.

From comparison of Figure 5a, with Figure 5b, where the water content is almost constant we see that the conductivity at a fixed temperature increases significantly with NaPSS content. Analogous trends have been also reported in References [26,28]. Figure 5b,c demonstrate for a fixed composition that the isothermal conductivity also increases significantly with water content.

The spectral shapes as well as the influence of temperature seen in the spectra are very similar to those of other ion-conducting materials. The conductivity spectra recorded at intermediate and high frequencies provide important insights into the ion motion. The dc conductivity reflects macroscopic transport, whereas the data at higher frequencies are determined by ionic motions on shorter time and length scales. In the following section, we will first discuss the dc conductivity and its activation enthalpy and then move to the discussion of the more localized dynamics probed in the dispersive regime of the conductivity spectra.

### 3.2. Arrhenius Representations of the DC Conductivity

**Figure 6:**
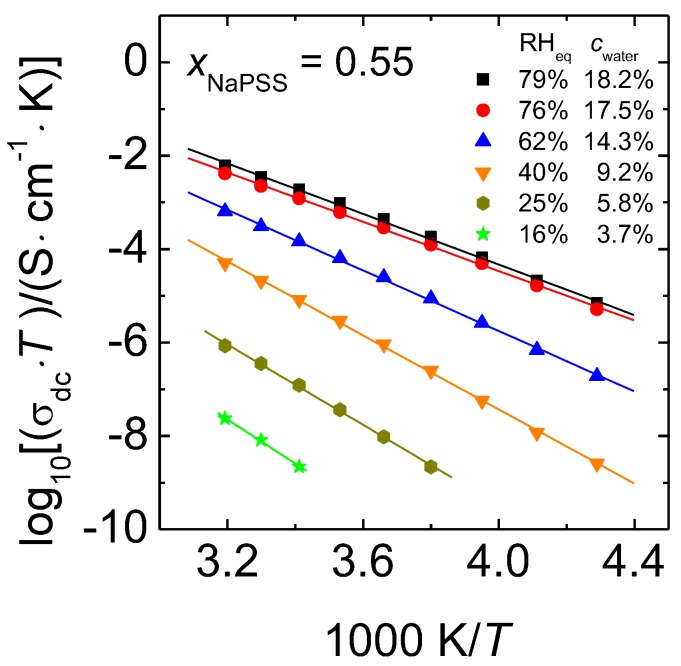
Arrhenius representations of data sets of 0.55 NaPSS∙0.45 PDADMAC PEC determined at different water contents. The error of the data is within symbol size.

The validity of the Arrhenius Equation was shown for completely dry PEC in Reference [26,32]. Arrhenius representations of the dc conductivity for hydrated PEC with *x*_NaPSS_ = 0.55 are displayed in Figure 6.

Following the Nernst-Einstein Equation, which yields a proportionality between the diffusion coefficient and $\sigma_{\mathrm{dc}}\cdot T,$ we have plotted $\;\log\left(\sigma_{\mathrm{dc}}\cdot T\right)$ on the *y*-axis. Here, we show for the first time that in all of the cases the dc conductivity of humid PEC follows the Arrhenius law.

(2) $${\;\sigma }_{\mathrm{dc}}\cdot T=A_{\mathrm{dc}}\cdot \exp\left[-\frac{∆H_{\mathrm{dc}}}{k_{B}\cdot \;T}\right]\;.$$

**Figure 7:**
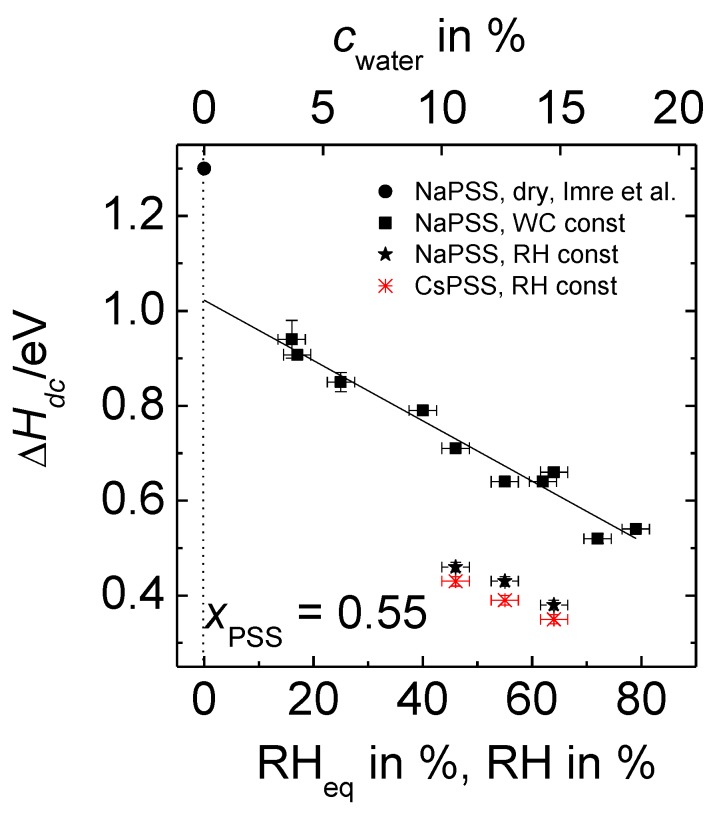
Activation enthalpies of *x* MPSS∙(1 − *x*) PDADMAC with *x*_PSS_ = 0.55 obtained at constant water content (squares) and at constant relative humidity (stars) in dependence on RH_eq_ or RH, respectively. The water content of the PEC in % (upper *x*-axis) corresponds to RH_eq_ of the constant water content (cWC) series (lower-*x*-axis). Data at constant RH have been determined for PEC with NaPSS (filled stars) and CsPSS (stars), respectively. The circle refers to completely dried PEC and has been taken from Reference [26].

In Equation (2), $∆H_{\mathrm{dc}}$ stands for the activation enthalpy of the ion transport determined for humid PEC samples, $A_{\mathrm{dc}}$ is the pre-exponential factor and $k_{B}$ the Boltzmann constant. As the focus of this work is on the temperature dependence of the conductivity and its activation enthalpy, entropic contributions to the conductivity, which are included in the exponential prefactor, will not be considered here further. The straight lines in Figure 6 were obtained by linear regression. Our data for PEC with constant water content show that the slope of the straight lines (which correspond to the activation enthalpy) increases with decreasing RH_eq_. In other words, the difference between the isothermal conductivities of samples with different water contents increases with decreasing temperature. Analogous trends are also found in the Arrhenius plots of data for the cRH-series, see Figure S3 (Supplementary Materials). The activation enthalpies determined from the data of Figure 6, Figure S3a,b are summarized in Figure 7. The *x*-labels RH_eq_ and *c*_water_ correspond to the measurement series with constant water content, whereas the label RH corresponds to data taken at constant RH.

The activation enthalpies determined at constant water content (filled squares) decrease linearly with increasing RH_eq_ or *c*_water_.

For comparison, the activation enthalpy for completely dried PEC from Reference [26] is also included (filled circle) into Figure 7. Note that the activation enthalpies even at our lowest investigated humidities are considerably smaller than those reported [26] for the dried PEC, viz. 1.31 eV for *x*_NaPSS_ = 0.55. Extrapolation of our data presented in Figure 7 to RH_eq_ = 0% yields Δ*H*_dc_ = 1.02 eV. One might speculate that the further lowering of RH_eq_ below 16% could result in a sharp increase of the activation enthalpy, which finally reaches the value of the completely dry case, implying strong deviations from the straight line presented in Figure 7. However, at which RH_eq_ value and to which extent such a change might be visible is currently still an open question. Possible deviations from the plotted linear relation could be related to structural changes of the PEC matrix, as well as with changes in the mechanism governing the ion dynamics. This important finding of a significant deviation between the measured and the extrapolated value of the activation enthalpy for dried PEC will be addressed in more detail in the section “Discussion”.

The activation enthalpies for the cWC-series, also shown in Figure 7, will be discussed in context with other physical quantities of this series in Section 3.4.

### 3.3. Scaling Behavior

In this subsection, we will analyze the conductivity spectra probing the ion dynamics on different time and length scales. Earlier scaling studies on hydrated PEC exposed to different RH values at a fixed temperature showed that the amount of absorbed water does not influence the number density of mobile ions. Instead, only the ion mobility is changed by water absorption [29]. Here we focus now on a scaling study where the temperature is changed while the water content in the PEC is kept constant. To the best of our knowledge, a scaling study of temperature-dependent conductivity spectra of hydrated PEC has never been performed before.

For a variety of materials including semiconductors [33,34], fullerenes [35], inorganic crystals [36] and glasses [37,38], ionic liquids [39], and polymeric systems [27], it has been shown that the shape of the conductivity spectra is independent of temperature. By appropriate normalization of the axes, the different conductivity isotherms can be therefore superimposed onto a master curve [40,41]. This is called “time-temperature-superposition-principle” (TTSP). The validity of the TTSP implies that the basic mechanism of transport does not change with temperature; instead, the dynamics on all of the time scales is uniformly accelerated with increasing temperature. The validity of the TTSP for the real part of the complex conductivity σ′/σ_dc_ can be expressed by a function σ′/σ_dc_ = F(ν/ν_0_) in which ν_0_ is an individual scaling parameter for each conductivity isotherm. The scaling function, F, is independent of temperature. An appropriate choice of ν_0_ for each curve is necessary to superimpose spectra measured at different *T* to a master curve. Summerfield scaling, often found to be valid for ion-conducting materials [33,34] can be considered as a special type of the more general TTSP and is fulfilled when ${\;\sigma }_{\mathrm{dc}}\cdot T$ and ν_0_ are proportional to each other. Summerfield scaling can therefore be expressed by

(3) $$\frac{\sigma \prime \left(v,T\right)}{\sigma_{\mathrm{dc}}\left(T\right)}=F\left(\frac{v}{\sigma_{\mathrm{dc}}\left(T\right)\cdot T}\right)\;.\;$$

**Figure 8:**
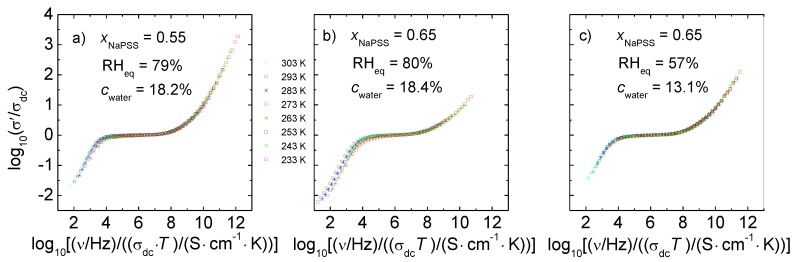
Conductivity spectra of Figure 5a–c scaled according to Summerfield scaling. All spectra of Figure 8 (**a**–**c**) were determined at constant water content as specified.

Figure 8 shows the spectra of Figure 5, but scaled according to the Summerfield concept. Indeed, here (and also for data taken at other compositions and water contents) we find that the Summerfield scaling is applicable. Additionally, spectra taken at constant RH are also in good agreement with this scaling approach, see examples in Figure S4 (Supplementary Materials).

The validity of the time-temperature superposition principle implies that in humidified PEC the basic conductivity mechanism does not change with temperature. Ionic motions on short length and time scales as well macroscopic transport on long timescales are accelerated simultaneously with an increasing temperature. Furthermore, the applicability of the Summerfield scaling approach shows that neither the number density of mobile ions nor the number of available ion sites change with temperature. Only the ion mobility increases with temperature.

Finally, it should be noted that the scaling relations of the *T*-dependent spectra of humidified PEC found in this work are in contrast to earlier results obtained for dried PEC. For dried *x* NaPSS∙(1 − *x*) PDADMAC PEC, deviations from Summerfield scaling were reported by Imre et al. [27].

### 3.4. Comparison with Data Determined at Constant Relative Humidity

The interpretation of data determined at fixed relative humidity is not straight forward because the water content in the PEC varies with temperature. In other words, the data taken at constant RH are always influenced by a combined effect of temperature and water content. Nevertheless, the results obtained in the cRH-series also contribute to the understanding of the ion transport mechanism in humid PEC, especially as we can compare the influence of the type of alkali cations present in the PEC.

Figure S1a (Supplementary Materials), which summarizes the water content in PEC as a function of the RH of the environment, is a presentation analogous to Figure 2, however for PEC with CsPSS instead of NaPSS. For PEC containing CsPSS, the proportionality factor of Equation (1) is 0.203 ± 0.003, indicating that the water content in PEC with CsPSS is always lower than for the corresponding PEC with NaPSS.

The conductivity data of the cRH-series show similarities to the results of the cWC-series. The general shape of the spectra is for example very similar to the data of the cWC series, see Figure S2 (Supplementary Materials) and Figure 5, respectively. We find the same spectral regimes as discussed before: a low-frequency regime determined by electrode polarization effects, a dc regime and a dispersive regime at high frequencies. At constant frequency the conductivity increases with temperature and RH.

Also, in the Arrhenius representations shown in Figure S3 (Supplementary Materials) we find analogous trends as for the data of the cWC-series in Figure 6. The accessible temperature and RH ranges are however more limited than in the cWC case.

When looking at the activation enthalpies determined from Figure S3a,b, which are also included in Figure 7, it is obvious that for NaPSS-PEC the activation enthalpies determined at constant water content are higher than for those determined at constant RH. For the cRH-series we have to take into account that the absolute amount of water in the surrounding atmosphere increases with temperature, and that therefore, the water content in the PEC is higher and the activation enthalpy lower than in the PEC investigated in the cWC-series. However, the limited *T*- and RH-range of the cRH-study, where only three activation enthalpies could be determined, does not allow for more detailed conclusions,

But not only the dc conductivities in the cRH- and the cWC-studies are similarly influenced by temperature variation. The influence of temperature on the conductivity spectra of the cRH-series is also similar as in the cWC-study. Figure S4a,b show the spectra of Figure S2a,b with scaled axes. All spectra in the investigated *T*-range could be superimposed onto a master curve using Summerfield scaling. This implies that also in PEC measured at constant RH, the number density of mobile ions or ionic sites do not vary with temperature and that temperature influences the ionic movements on different time and length scales in the same way.

In all of the investigated systems (cRH-series and cWC-series) we also find that the conductivity increases with PSS content. An increase of the conductivity of PEC with PSS content has been published before [26,28,32]. This increase was assigned to an increasing number density of mobile cations as well as to an increasing ion mobility. The latter is due to the fact that with an increasing amount of mobile ions, the number of possible ion sites increases as well yielding a network of better connected pathways for the ion transport [26].

For the cRH-series we also see differences between the PEC, which either contain Cs^+^ or Na^+^ ions. Such differences have already been discussed for room temperature data in Reference [28]. Nevertheless, the temperature-dependent studies presented here give more insight into the transport mechanism of the ion movements. A comparison of the four Figure S3a–d (Supplementary Materials) reveals that at a given temperature the conductivity is higher in *x* CsPSS∙(1 − *x*) PDADMAC than in *x* NaPSS∙(1 − *x*) PDADMAC.

Additionally, at constant RH the PEC with CsPSS not only show higher dc conductivities but they also have lower activation enthalpies than those with NaPSS. These findings strengthen the conclusion put forward in References [28,29], viz. that in humid PEC with *x*_PSS_ > 0.5, Cs^+^ ions move faster than Na^+^ ions. As the amount of small counterions is defined by the PEC composition, which is the same for PEC with Na^+^ and Cs^+^, the reason for the different mobilities might be due to a varying size of the water shells for both types of cations. Comparison of Figure 2 and Figure S1 clearly shows that PEC prepared from CsPSS absorb less water than those with NaPSS. Na^+^ being a smaller ion as compared to Cs^+^ has a higher charge density (*q*/*r*, where *q* is the charge of the ion and *r* is the ion radius) than Cs^+^. Consequently, Na^+^ ions possess a larger hydration shell as compared to Cs^+^ ion. This conclusion is in accordance with the radius of hydrated alkali ions as published in Reference [42]. Another reason for the higher mobility of the Cs^+^ ions might be that according to Reference [42] larger alkali ions bind the immediately adjacent water molecules only weakly, allowing a partial dehydration of the moving ions. One might argue that the higher conductivity of PEC with cesium ions arises from a higher dissociation constant for CsPSS as compared to NaPSS. However, if that was the case, the degree of dissociation should be affected by a variation of humidity/water content and temperature, respectively. This would imply that the number density of mobile ions of a given system was not constant. However, in that case, deviations from Summerfield scaling should occur, which is not the case. The finding that in hydrated PEC the mobility increases with cation radius is in agreement with previous findings [28] and in vast contrast to completely dried PEC where the bare ions carry the charge [32].

## 4. Discussion

The major findings we obtain on the basis of our systematic temperature-dependent conductivity study at fixed relative humidity and at fixed water content are as follows:

1. Under same external conditions PEC with NaPSS absorb more water than PEC with CsPSS.
2. At fixed temperature and humidity, CsPEC show a higher conductivity than NaPEC.
3. The dc conductivity increases as a function of temperature, humidity and/or water content.
4. The dc conductivity obtained at constant water content and at constant RH increases with increasing PSS content.
5. The temperature dependence of the dc conductivities of all humid PEC follows the Arrhenius law.
6. The activation enthalpy values for humid PEC are significantly lower than those of completely dried complexes as reported in Reference [26]. For the composition and temperature range investigated, the activation enthalpies determined at fixed relative humidity are lower than those determined at fixed water content. Moreover, in case of fixed humidity measurements, PEC prepared from CsPSS possess lower activation enthalpies than PEC with NaPSS under same temperature and humidity condition.
7. The time-temperature superposition principle is valid for the conductivity spectra of humidified PEC and the conductivity spectra can be scaled with the help of the Summerfield scaling.

These findings together allow for detailed conclusions about the ion transport in PEC. On the basis of room temperature conductivity studies, De et al. have already shown that water molecules absorbed in humid PEC not only hydrate the ions, but also facilitate the ion motion via screening the electrostatic interactions between polyion-polyion and polyion-cation, and thereby lowering the barriers in the energy landscape of the migrating ions [28,29]. This reduction of electrostatic interactions increases with RH and causes a stronger enhancement of the ionic mobility by softening of the polymer matrix. In References [28,29], it was postulated on the basis of room-temperature conductivity data that the presence of water softens the complete PEC network and reduces activation barriers for the ion transport. The new *T*-dependent conductivity measurements in both of our measurement series presented here, now provide the first direct evidence that the presence of water molecules indeed reduces the activation enthalpies.

Assuming that water exists in two different sites, i.e., hydration water of the ions and water molecules absorbed by PEC that do not participate in hydration of the counterions (matrix water), we can use the above findings to interpret our temperature-dependent data. The activation enthalpies reported here show that the matrix water molecules lower the energy barriers for the moving ion/hydration shell complexes. This effect gets stronger with increasing humidity at which the samples were equilibrated, as shown in Figure 6. We can now make distinct conclusions about this lowering effect.

In Reference [29], which was based on RH-dependent conductivities measured at ambient temperature, we had suggested the following relation to be valid:

(4) $$\sigma_{\mathrm{dc}}\left(\mathrm{RH},T\right)\cdot T\;\propto \;\exp\left[-\frac{\left(∆H_{\mathrm{dc},\mathrm{dry}}-B^{\#}\cdot \mathrm{RH}\right)}{k_{B}\;\cdot T}\right]\;\mathrm{with}\;B^{\#}=B\cdot k_{B}\cdot \;T\;.\;$$

Here, $∆H_{\mathrm{dc},\mathrm{dry}}$ stands for the activation enthalpy of the dried complexes. It should be noted that in contrast to Reference [29], we have here added the index “dry” to avoid confusion with the $∆H_{\mathrm{dc}}$ data presented in this work. The parameter $B^{\#}$ can be determined from the slope *B* that is obtained by linear regression of ln$\left(\sigma_{\mathrm{dc}}\left(\mathrm{RH}\right)\cdot T\right)$ data versus RH [28,29], see also Figure 4. As discussed before, *B* depends linearly on 1/*T* implying that $B^{\#}$ is independent of temperature. With the analysis of our *T*-dependent spectra for humid PEC with constant water content, we can now for the first time confirm the reduction in the activation enthalpy for the ion transport. Figure 7 shows that there is in fact a linear relation between $∆H_{\mathrm{dc}}$ and the relative humidity for both types of series, as expected from Equation (4). $∆H_{\mathrm{dc}}$ should be equal to $∆H_{\mathrm{dc},\mathrm{dry}}-B^{\#}\cdot \mathrm{RH}$. However, the extrapolation of the data in Figure 7 for NaPSS–PEC to RH = 0% shows that $∆H_{\mathrm{dc},0\%}$ does not match with $∆H_{\mathrm{dc},\mathrm{dry}}$ where a value of 1.31 eV was found [26]. The $∆H_{\mathrm{dc},0\%}$ value of 1.02 eV obtained in the present study from extrapolation for constant water content is much lower than expected from a completely dried complex. Therefore, Equation (4) has to be modified in one respect: $∆H_{\mathrm{dc},\mathrm{dry}}-B^{\#}\cdot \mathrm{RH}$ has to be replaced by $∆H_{\mathrm{dc},0\%}-B^{\#}\cdot {\mathrm{RH}}_{\mathrm{eq}}$, so that we obtain:

(5) $$\sigma_{\mathrm{dc}}\left(\mathrm{RH},T\right)\cdot T\;\exp\left[-\frac{(∆H_{\mathrm{dc},0\%}-B^{\#}\cdot {\mathrm{RH}}_{\mathrm{eq}}}{k_{B}\;\cdot T}\right]\;\mathrm{with}\;B^{\#}=B\cdot k_{B}\cdot \;T\;.\;$$

Alternatively, the water content *c*_water_, which is proportional to ${\mathrm{RH}}_{\mathrm{eq}}$ could be used in Equation (5) instead of ${\mathrm{RH}}_{\mathrm{eq}}.$

The microscopic origin for the lowering of activation enthalpy could be explained by the fact that due to absorption of water by PEC at elevated relative humidity, the activation barriers for the ion transport are lowered. The lowering effect of activation enthalpy is more prominent with an increasing relative humidity. The difference between $∆H_{\mathrm{dc},0\%}\;$obtained from extrapolation of the humid PEC and $∆H_{\mathrm{dc},\mathrm{dry}}$ implies that the polymer network and the charge transport properties of completely dried PEC seem to differ significantly from those of humid PEC. This is due to the fact that humidity softens the polymer matrix and reduces the electrostatic interactions between polyion-polyion and polyion-cation, and thereby lowering the barriers in the energy landscape of the ion migration. Such lowering effects are absent in the dry complexes.

Another significant difference between humid and dried PEC is also seen in the scaling relations for the respective temperature-dependent conductivity spectra. In both cases scaling is possible, which shows that the time-temperature-superposition principle is fulfilled for dried and hydrated PEC. However, in contrast to humidified PEC where Summerfield scaling is applicable, in dried PEC of the same composition, Baranowskii/Cordes scaling [27,43] was employed. The latter type of scaling includes an additional scaling factor for the frequency scale, which is *T*-dependent. For dried PEC two possible reasons were given to explain the deviations from Summerfield scaling for PEC with an excess of PSS [27]. Either the number density of mobile alkali ions released from their countercharges increases with temperature or the number of available pathways for the ion transport decreases with *T*. The latter effect can be caused by temperature-induced changes of the chain conformation or a higher local mobility of chain segments in the polyelectrolyte network resulting in a blocking of some pathways for the Na^+^ ion transport.

By contrast, the validity of Summerfield scaling in hydrated *P*EC implies that the number density of ions or ionic sites does not change with temperature. Additionally, the mechanism of ion movements on local scales (probed by the dispersive conductivity above the dc plateaus) as well as macroscopic transport (probed by the dc plateau) is not influenced by temperature. The effect of temperature is simply to be seen as an acceleration of the ion dynamics. The reason why we observe Summerfield scaling in humid PEC might be due to the fact that the absorbed water releases all of the alkali ions present in the PEC, even at lower temperatures. Therefore, no increase in the number density of mobile ions is detected with temperature. In addition, the absorbed water further softens the complete PEC network which leads to generally faster dynamics resulting from structural rearrangements. This could be the reason why no pathways for the ion transport are blocked when the temperature is changed.

The scaling results on PEC with constant RH presented in this work also strengthen the conclusions of References [28,29], that in hydrated PEC, alkali ions move with their hydration shells and that the conductivity is not governed by proton transport:

1. If proton transport dominated the dc conductivity, one would expect that the conductivity in the cRH-series should increase with temperature because of the additional absorption of water into the PEC.
2. An increasing water content in the PEC should go hand in hand with an increasing number of mobile charge carries, which would result in deviations from the Summerfield scaling concept. By contrast, the number density of mobile ions is not *T*-dependent, indicating that hydrated alkali ions are the major charge carriers.
3. PEC prepared from CsPSS show under same conditions a higher conductivity than PEC with NaPSS. This also confirms that the alkali ions in their hydrations shell determine the ion transport properties.

This work has focused on PEC, which have an excess of PSS and therefore alkali ions. It would now be interesting to analyze the ion dynamics in humid PEC with an excess of PDADMAC and therefore of Cl^−^ ions. This will be topic of a forthcoming paper.

## Supplementary Materials

The following are available online at www.mdpi.com/2073-4360/9/11/550/s1. Figure S1: (a) Influence of the relative humidity of the environment on the water content in *x* CsPSS∙(1 − *x*) PDADMAC at ambient temperature (b) Water content of 0.55 MPSS∙0.45 PDADMAC PEC with Na+ and Cs+ ions, respectively. All straight lines have been calculated using Equation (1). In the case of PEC with NaPSS the proportionality factor is 0.230 ± 0.005, whereas it is 0.203 ± 0.003 for PEC with CsPSS; Figure S2: Temperature-dependent conductivity spectra of the cRH-series: (a) NaPEC and (b) CsPEC, both with MPSS = 0.60. At each temperature the samples were kept in an environment with 46% RH; Figure S3: Arrhenius plots of the PEC with MPSS from the cRH-series. The data were taken at three different RH values, viz. 46%, 55% and 64%. Data for different compositions and types of alkali cations are shown: (a) *x* NaPSS = 0.55, (b) *x* CsPSS = 0.55, (c) *x* NaPSS = 0.60, (d) *x* CsPSS = 0.60; Figure S4: Master curves obtained by Summerfield scaling of the different isotherms displayed in Figure S2 (a) PEC with *x* NaPSS = 0.60 and (b) PEC with x CsPSS = 0.60. The samples were exposed to RH = 46% at each temperature (cRH-series).
