# Supplementary material for: Ion Conduction and Its Activation in Hydrated Solid Polyelectrolyte Complexes
Source: Polymers (Basel). 2017 Oct 25;9(11):550. doi: 10.3390/polym9110550 (PMC6418868; doi:10.3390/polym9110550)
Supplement: Supplementary file 1 [file polymers-09-00550-s001.pdf]

Supplementary Materials

Ion Conduction and its Activation in Hydrated Solid Polyelectrolyte Complexes

Souvik De [1,2,†], Annika Ostendorf [2,†], Monika Schönhoff [2] and Cornelia Cramer [2,*]

[1] NRW Graduate School of Chemistry, University of Muenster, Wilhelm-Klemm-Str. 10, D-48149 Muenster, Germany; souvikiitm@gmail.com

[2] Institute of Physical Chemistry, University of Muenster, Corrensstraße 28/30, D-48149 Münster, Germany; Annika-Ostendorf@gmx.de (A.O.); schonhoff@uni-muenster.de (M.S.)

* Correspondence: cramerc@uni-muenster.de; Tel.: +49-251-832-3412

† These authors contributed equally to this work.

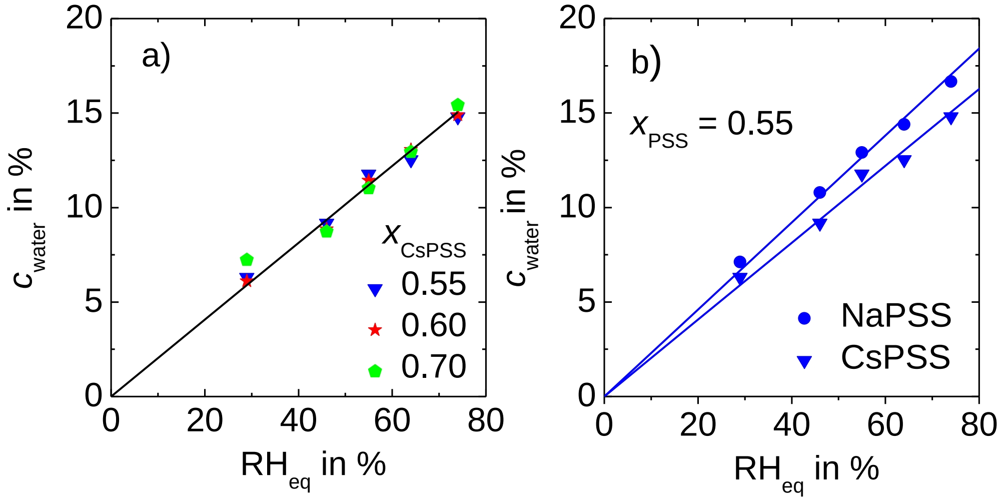

S1: (a) Influence of the relative humidity of the environment on the water content in *x* CsPSS·(1-*x*) PDADMAC at ambient temperature.

(b) Water content of 0.55 MPSS · 0.45 PDADMAC PEC with Na^+ and Cs^+ ions, respectively. All straight lines have been calculated using Equation (1). In the case of PEC with NaPSS the proportionality factor is 0.230 ± 0.005, whereas it is 0.203 ± 0.003 for PEC with CsPSS.

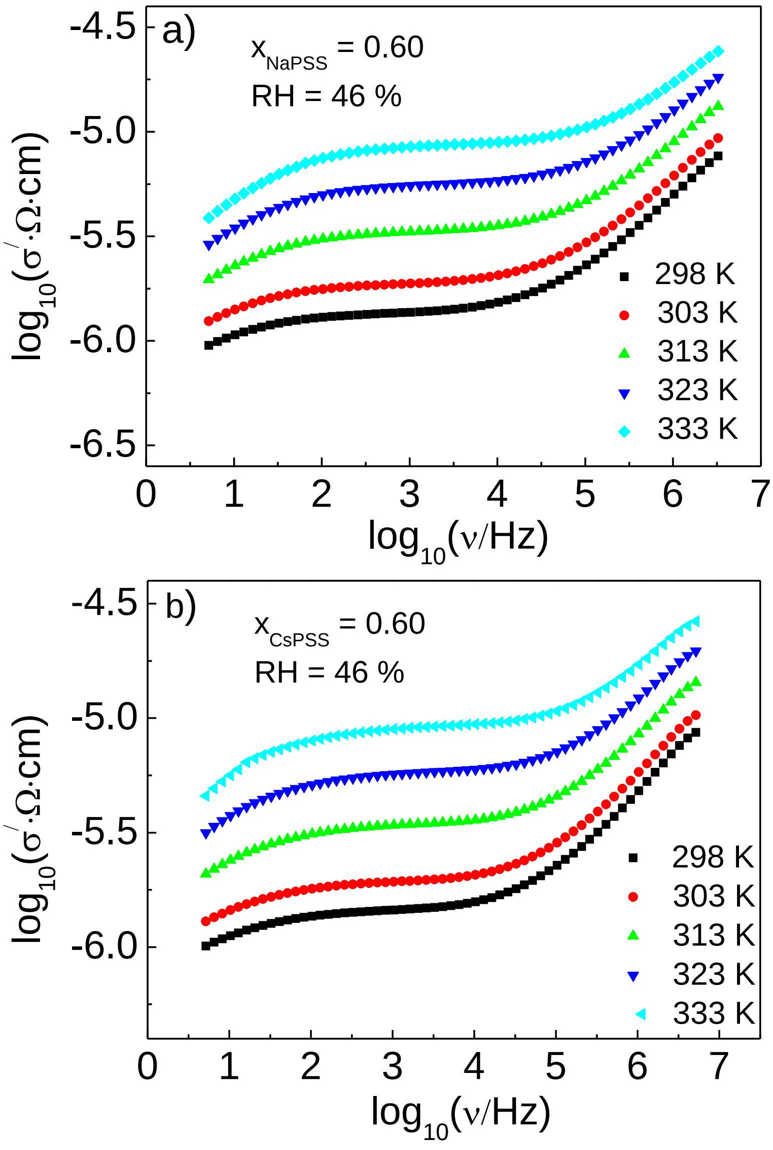

S2: Temperature-dependent conductivity spectra of the cRH-series: (a) NaPEC and (b) CsPEC, both with MPSS = 0.60. At each temperature the samples were kept in an environment with 46% RH.

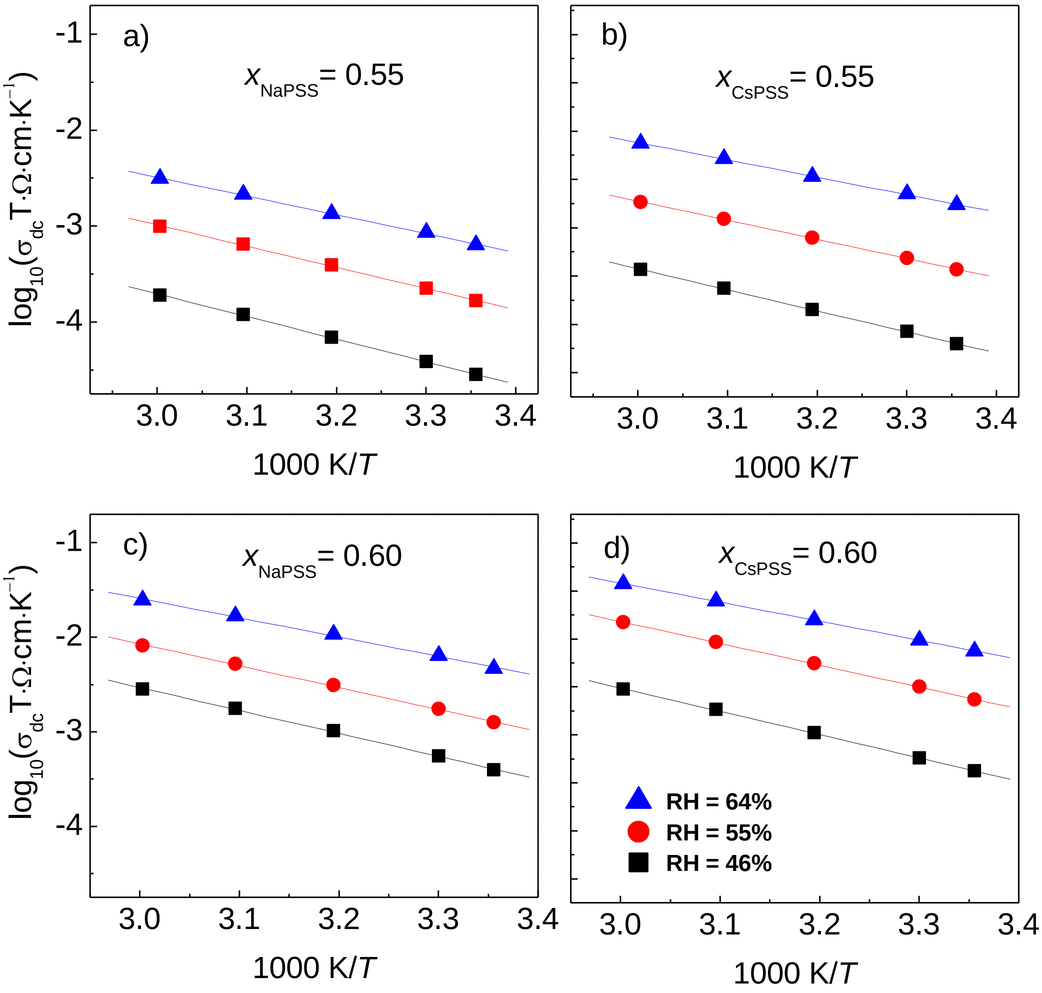

S3: **Arrhenius plots of the PEC with MPSS from the cRH-series.**
The data were taken at three different RH values, viz. 46%, 55% and 64%. Data for different compositions and types of alkali cations are shown: (a) x_{NaPSS} = 0.55, (b) x_{CsPSS} = 0.55, (c) x_{NaPSS} = 0.60, (d) x_{CsPSS}= 0.60.

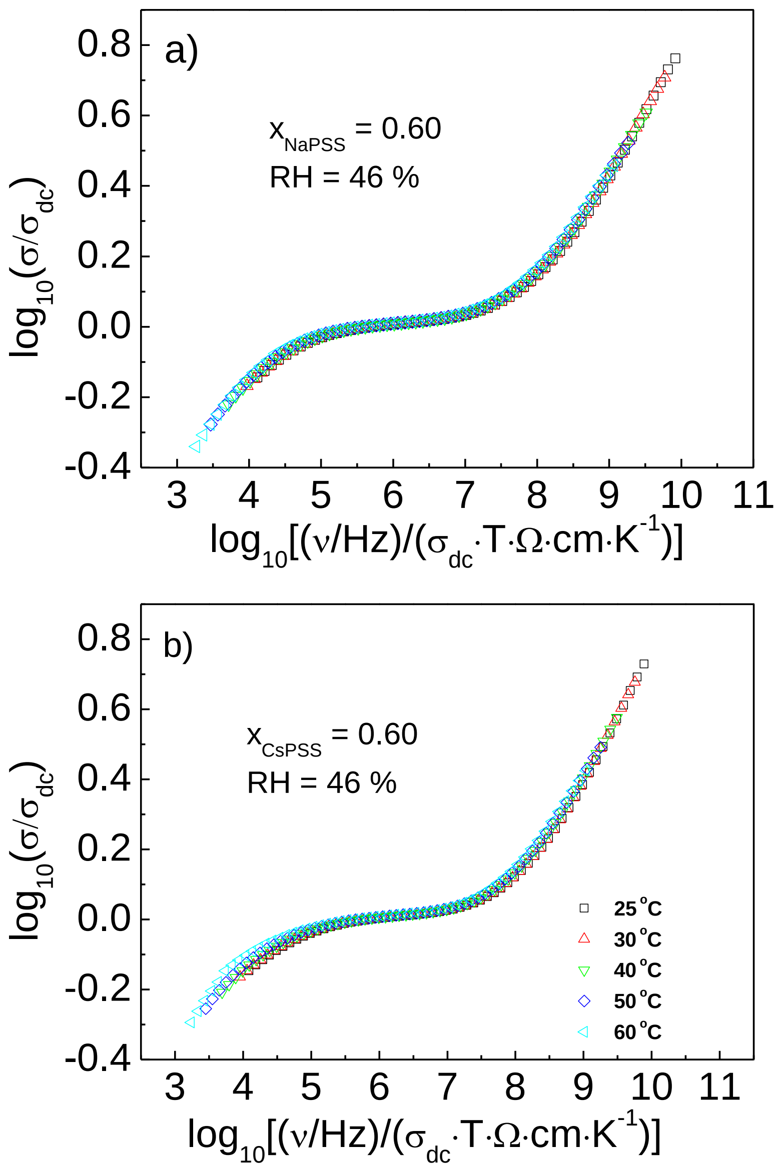

S4: **Master curves obtained by Summerfield scaling of the different isotherms displayed in Figure S2**

(a) PEC with x_{NaPSS} = 0.60 and (b) PEC with x_{CsPSS} = 0.60. The samples were exposed to RH = 46% at each temperature (cRH-series).
